# Insect-specific viruses: transmission dynamics and biological control strategies against arboviruses

**DOI:** 10.3389/fmicb.2025.1624662

**Published:** 2025-08-29

**Authors:** Lukas Wilkman, Elin Kaarle, Verah Nafula Luande, Rebecca Lantto, Magnus Evander, Olivia Wesula Lwande

**Affiliations:** ^1^Department of Clinical Microbiology, Umeå University, Umeå, Sweden; ^2^Department of Epidemiology and Global Health, Umeå, Sweden; ^3^Umeå Centre for Microbial Research (UCMR), Umeå, Sweden

**Keywords:** insect-specific virus, *Flaviviridae*, *Peribunyaviridae*, *Togaviridae*, arbovirus, mosquito, biological control

## Abstract

Mosquitoes are known to vector arthropod-borne viruses (arboviruses) that pose a global public health issue in the form of mosquito-borne viral diseases such as chikungunya fever, dengue fever, Japanese encephalitis, yellow fever, and Zika. Besides, mosquitoes may also carry insect-specific viruses (ISVs), which are evolutionarily alike arboviruses yet do not infect vertebrates. These ISVs have been shown to affect the ability of mosquitoes to transmit arboviruses, as well as potentially inhibit arbovirus infections in vertebrate hosts. Yet, ISVs still constitute a relatively new and little-researched area where further studies may yield new knowledge regarding their distribution, their future importance in the control of mosquito-borne viral disease and potential role in biological control of mosquitoes. This review provides insights into ISV classification, transmission, and biology, as well as historical and future aspects. It mainly focuses on the characterization of the transmission dynamics of ISVs to highlight the various potential arboviral pathogen transmission blocking mechanisms along with evolution and host tropism. The review also provides additional information on the potential use of ISVs as a method of biological control in comparison to other proposed methods as well as delving into current research into arbovirus-based vaccines and antiviral drug development.

## Introduction

Arthropod-borne virus (arbovirus) is the collective term for viruses spread by arthropods. Globally, hundreds of millions of people are affected by diseases caused by arboviruses (Girard et al., 2020). Mosquitoes are the key vectors for many arboviruses causing human disease that constitute a major threat to global public health. In response to the risk of arbovirus epidemics and potential pandemics, WHO announced the Global Arbovirus Initiative in 2021, providing a list of priority actions to prevent future arbovirus outbreaks (Balakrishnan, 2022). These viral diseases include dengue fever, chikungunya fever, o’nyong-nyong fever, West Nile fever, Zika virus disease and many others (Siew et al., 2025). For this reason, arbovirus surveillance in diverse mosquito species is of great importance. Mosquito surveillance may predict and help prevent larger outbreaks of viral diseases (Engdahl et al., 2014).

In addition to viruses causing human disease, mosquitoes may also carry insect-specific viruses (ISVs) (Blitvich and Firth, 2015; Vasilakis and Tesh, 2015; Agboli et al., 2019). These viruses are unable to replicate in vertebrate cells and are therefore unable to cause infection in humans. First discovered 40 years ago, ISVs have garnered increasing interest in recent times as their interactions with human arboviruses have been further studied and their potential use in limiting the disease burden of human arbovirus infections recognized (Stollar and Thomas, 1975; Bolling et al., 2012; Patterson et al., 2020; Carvalho and Long, 2021). Globally present, ISVs have been isolated from mosquitoes on all continents where mosquitoes are present (Huhtamo et al., 2009; Moonen et al., 2023).

This review will explore the classification of these mosquito-borne viruses as well as the historical perspectives of both mosquito-borne arboviruses and the discoveries of insect-specific viruses, while also highlighting current research into the transmission dynamics, evolution, and tropism of ISVs in the study of arbovirus transmission and infections, as well as their use in the development on new methods of biological control and vaccines.

## Classification of mosquito-borne viruses

**Figure 1:**
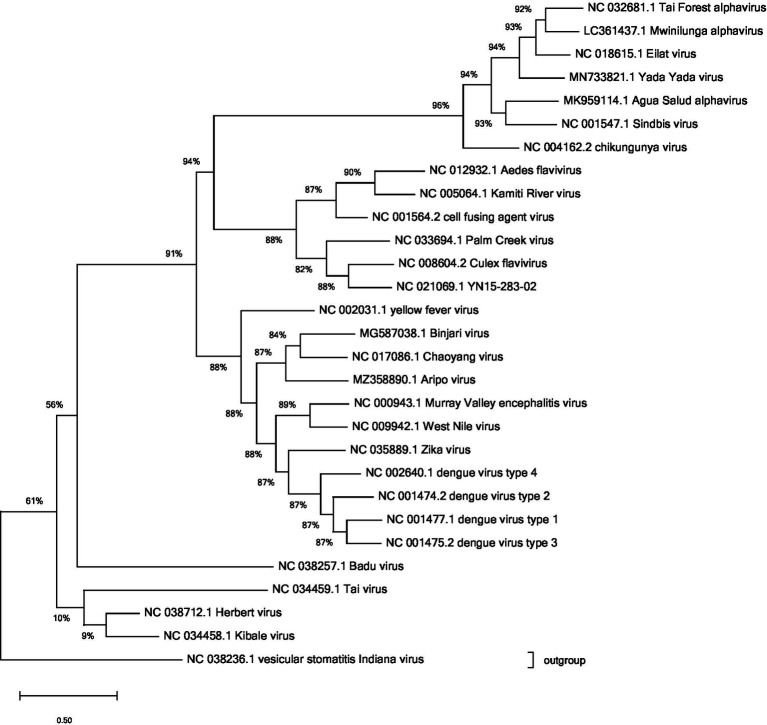
Phylogenetic analysis of reference sequences for the arboviruses and ISVs discussed. The families visualised are *Togaviridae* (chikungunya virus), *Flaviviridae* (dengue virus), and *Peribunyaviridae* (Badu virus). The typical member of *Rhabdoviridae* was used as an outgroup on which the tree was rooted. Data from NCBI virus. Alignment performed using ClustalW algorithm. Tree drawn using maximum likelihood in Mega 11 software. Where a reference sequence was not available (Mwinilunga virus, Agua Salud alphavirus and Yada Yada virus) the most complete sequence catalogued was used.

The majority of mosquito-borne viruses, including those causing human disease as well as ISVs, belong to one of the three major families of arboviruses: *Flaviviridae*, *Peribunyaviridae* or *Togaviridae*, see Figure 1 (Blitvich and Firth, 2015; Vasilakis and Tesh, 2015; de Almeida et al., 2021). In viewing Figure 1, it is of note that the lineage of insect-specific flaviviruses (ISFs) is genetically distinct from the other flaviviruses and is, as such, placed in a separate branch. The same cannot be observed for insect-specific viruses in the other families pictured. Some mosquito-borne viruses are found in the families *Phenuiviridae*, *Reoviridae* and *Rhabdoviridae* (Vasilakis and Tesh, 2015). Additional ISVs can be found in the families *Mesoniviridae*, *Tymoviridae*, *Birnaviridae* and *Iridoviridae,* among others (Carvalho and Long, 2021). Negevirus, an insect specific viral taxon has been described as well (Vasilakis et al., 2013).

The family *Flaviviridae* includes over 90 different viruses and of these, 39 are classified as arthropod-borne flaviviruses (Huhtamo et al., 2014; Postler et al., 2023). These mosquito-borne flaviviruses include both arboviruses, such as dengue virus (DENV) and yellow fever-virus (YFV), and many insect-specific viruses (ISVs) (Huhtamo et al., 2014). ISFs include cell fusing agent virus (CFAV), Kamiti river virus (KRV) and Culex flavivirus (CxFV) (Sang et al., 2003; Hoshino et al., 2007). ISFs are, as other flaviviruses, enveloped RNA-viruses with a positive sense single-stranded genome of approximately 9–13 kb (Huhtamo et al., 2014).

*Peribunyaviridae* is one of the largest families of RNA-viruses, including over 140 different viruses, the majority of which are arboviruses (Hughes et al., 2020). There are several diseases affecting humans caused by mosquito-borne *Peribunyaviruses* (Marklewitz et al., 2013), but the family also includes ISVs (Ballinger et al., 2014). The insect-specific *Peribunyaviruses* include Herbert virus (HEBV), Tai virus (TAIV), Kibale virus (KIBV), and the newly discovered Badu virus (BADUV) (Hobson-Peters et al., 2016). The viruses are enveloped RNA-viruses with three negative-sense single-stranded segments: S, M, and L, totaling approximately 10.7–12.5 kb (Marklewitz et al., 2013).

The family *Togaviridae* contains only the genus *Alphavirus* (Walker et al., 2019). The majority of alphaviruses are transmitted by mosquitoes to humans and other vertebrates (Chen et al., 2018). Alphaviruses causing human disease include chikungunya virus (CHIKV) and Sindbis virus (SINV) (Nasar et al., 2012) At present, five ISVs have been discovered in the family *Togaviridae*: Eilat virus (EILV), Tai Forest alphavirus (TALV), Mwinilunga alphavirus (MWAV), Agua Salud alphavirus (ASALV), and Yada Yada virus (YVV) (Nasar et al., 2012; Hermanns et al., 2017, 2020; Torii et al., 2018; Batovska et al., 2020). Viruses in the family *Togaviridae* are enveloped RNA-viruses with a positive-sense single-stranded genome of approximately 10–12 kb, and so have many structural similarities to the flaviviruses (Chen et al., 2018).

In addition, there are ISVs that do not belong to the major mosquito-borne families, for instance *Mesoniviridae*, negeviruses, *Reoviridae*, *Nodaviridae*, *Rhabdoviridae* have been detected (Bolling et al., 2015). For example, recently we discovered Hubei chryso-like virus, belonging to the family *Chrysoviridae* (Lwande et al., 2023). These viruses naturally represent a varied selection of evolutionary pathways. For instance, the negeviruses share a genetic similarity with plant viruses in the genera *Blunervirus, Cilevirus* and *Higrevirus* to the extent that plants have been suggested to be involved in the natural transmission pathways (Vasilakis et al., 2013; Nunes et al., 2017). The implications of this variation in evolutionary paths are regarding tropism are further discussed below.

## Historical perspective

In 1975, the first ISV was isolated from a cell culture of *Aedes aegypti* and was named cell-fusing agent virus (CFAV) for its characteristic cytopathic effect (CPE), where multinuclear cells are produced (Stollar and Thomas, 1975). When inoculated into vertebrate cells, the virus produces no CPE and cannot be re-isolated, signifying that the virus is specific to arthropod cells. Mosquitoes collected in Puerto Rico in 2002 were carrying CFAV, making the first discovery and isolation of CFAV in a natural mosquito population (Cook et al., 2006). Many ISVs have been discovered since 1975, especially during the last two decades with increasing mosquito surveillance and improved sequencing methods (Carvalho and Long, 2021). Of these ISVs, many have been shown to belong to the family *Flaviviridae*. ISVs have been isolated from many different species of mosquitoes and mosquito cell lines.

The first ISV related to CFAV, then long considered the only ISV, was found in Kenya in 1999 (Lutomiah et al., 2007). The virus was named Kamiti River virus (KRV) and belongs to the family *Flaviviridae* (Sang et al., 2003). The virus was found in wild-caught *Aedes macintoshi* mosquitoes, which for the first time demonstrated that ISVs were present in nature, not only in laboratory cell cultures. In a study by Crabtree et al., arthropod cells were infected with KRV and CFAV to compare CPE. Infection with KRV led to rounding of the cells, de-anchoring from the culture flask, and cell death. The main difference between KRV and CFAV is that CFAV causes massive syncytium formation, whereas KRV does not (Crabtree et al., 2003).

Another ISV was found in Japan, isolated from *Culex pipiens* mosquitoes collected in 2003, making it the first ISV isolated in *Culex* mosquitoes. The virus was named Culex flavivirus (CxFV), belonging to the family *Flaviviridae*. Culex flavivirus was later found in Japan, Indonesia, Guatemala, Mexico, USA, Trinidad and Tobago, and Vietnam (Hoshino et al., 2007). Phylogenetic analysis shows that CxFV is closely related to both CFAV and KRV. C6/36 cells, a cell line derived from *Aedes*-mosquitoes, display weak cytopathic effects (CPE) when infected with CxFV (Hoshino et al., 2007; Morales-Betoulle et al., 2008; Crabtree et al., 2009; Farfan-Ale et al., 2009; Kim et al., 2009). A second Japanese ISV was found in *Aedes albopictus*-mosquitoes. The virus is a member of the family *Flaviviridae* and was thus named Aedes flavivirus (AEFV). C6/36 cells infected with AEFV display weak CPE, though the effect is milder than the one caused by CxFV (Hoshino et al., 2009). In addition, an ISV belonging to the Togaviridae family-Eilat virus (EILV) was isolated from *Anopheles coustani* in Israel between 1982 and 1984 (Samina et al., 1986; Nasar et al., 2012). The virus has been shown to replicate in high titres in insect cell lines but interfere with transmission of pathogenic alphaviruses such as CHIKV, Western Equine Encephalitis virus (WEEV), Venezuelan Equine Encephalitis virus (VEEV) and Eastern Equine Encephalitis virus (EEEV) (Nasar et al., 2015). Further discoveries are continuously being made, with new ISVs being categorized.

### Transmission dynamics for ISVs

**Figure 2:**
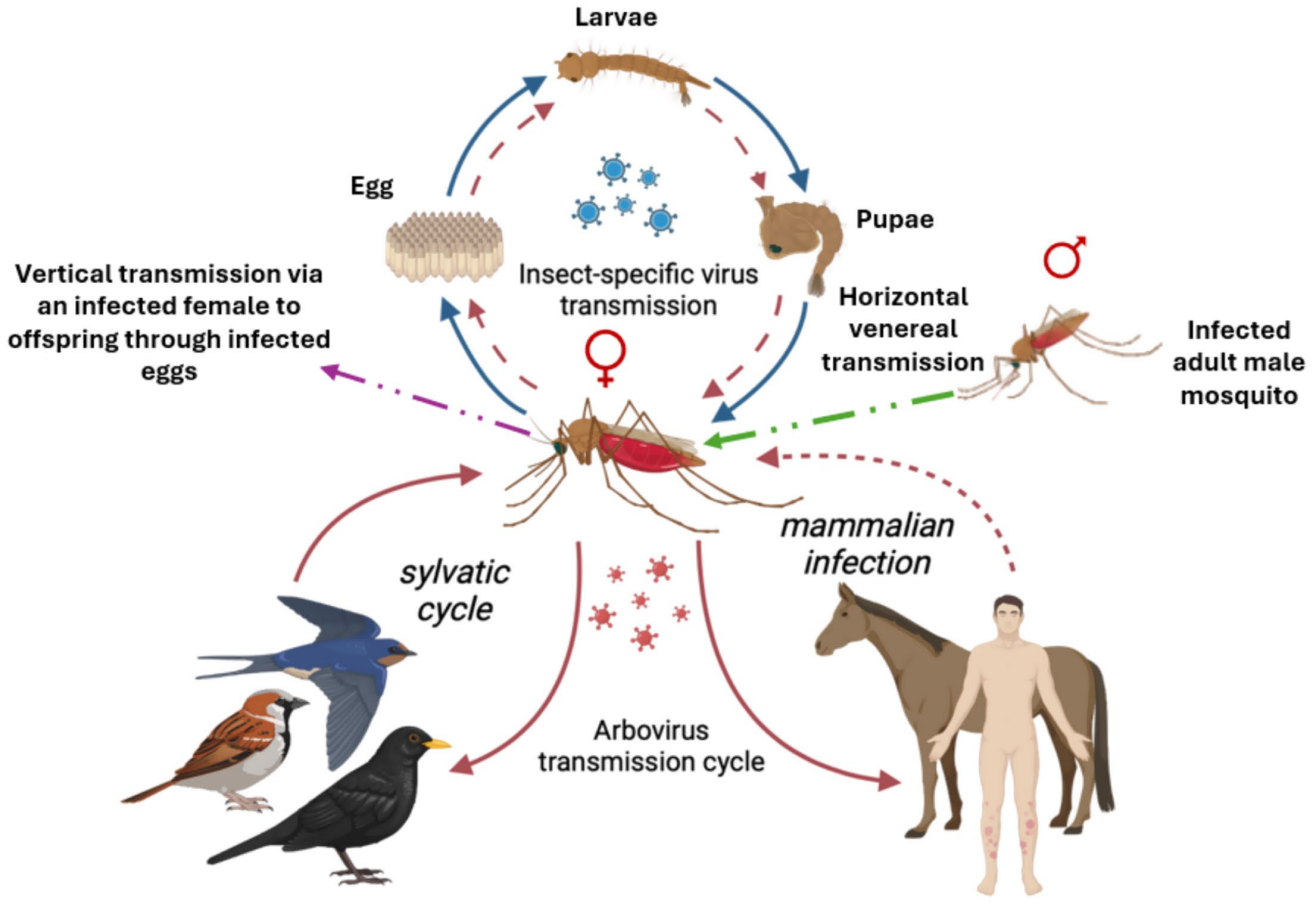
Transmission dynamics for insect specific viruses (blue) and arboviruses (red). Arboviruses often form a sylvatic, enzootic cycle in their environment, often with smaller vertebrates such as avian host. Larger vertebrates, such as humans are often dead-end hosts, but exceptions are known (dotted line). Some arboviruses are also able to be vertically transmitted in the vector (dotted circle) Insect specific viruses, however, do not infect vertebrate hosts, as such, only vertical transmission through an infected adult female mosquito to her progeny through eggs (purple dotted arrow) as well as horizontally through venereal transmission during mating between infected and uninfected adult male and female mosquitoes (green dotted arrow), is depicted. Figure created with BioRender.com.

Insect-specific viruses (ISVs) are unique in their strict host range, infecting only insect vectors mainly mosquitoes, without the ability to infect vertebrate hosts. Understanding their transmission dynamics is essential for evaluating their potential role in vector control and arbovirus interference, hence blocking transmission. ISVs are primarily maintained within mosquito populations through vertical transmission, where infected female mosquitoes pass the virus directly to their progeny via infected eggs (Lutomiah et al., 2007; Zhang et al., 2017) (see Figure 2). This mode ensures long-term persistence, maintenance and stable circulation of ISVs within vector populations. Additionally, horizontal transmission occurs through venereal transmission during mating between infected and uninfected adult mosquitoes, further facilitating the spread of ISVs among adults (Wen et al., 2022; Peterson et al., 2024; Sharpe et al., 2024) (see Figure 2). Other possible routes include oral transmission during larval stages or through co-feeding behavior, though these mechanisms are less well characterised and require further validation (Blitvich and Firth, 2015). The efficient transmission of ISVs within mosquito populations enables their sustained presence without infecting vertebrates, positioning them as promising agents for biological control. However, the exact molecular and cellular mechanisms that restrict ISVs to insect hosts remain under scrutiny (Elrefaey et al., 2020). Elucidating these barriers will help clarify their safety profile and potential interactions with arboviruses, paving the way for their strategic application in interrupting mosquito-borne disease transmission.

Mosquitoes can carry both arboviruses and ISVs, see Figure 2 (Hobson-Peters et al., 2013; Blitvich and Firth, 2015; Vasilakis and Tesh, 2015; Agboli et al., 2019). Arboviruses can infect both arthropod- and vertebrate cells, while ISVs exclusively replicate in arthropod cells. These phenomena are called dual-host for arboviruses, and single-host for ISVs. Evolutionarily, arboviruses and ISVs appear similar in origin, suggesting that arboviruses may have been ISVs that evolved the capability to also infect vertebrate cells, expanding their host range (Blitvich and Firth, 2015). ISVs are most likely transmitted by vertical transmission, i.e., transovarially from an infected female to their offspring. The offspring of ISV-infected mosquitoes have been shown to be positive for the same virus the female carries for many ISVs, including CFAV, CxFV, and AEFV (Bolling et al., 2011; Haddow et al., 2013; Logan et al., 2022). Through vertical transmission ISVs can be spread through generations and are frequently found in sampled mosquito populations. As the viruses are unable to infect vertebrate cells, they cannot be further spread through infected females taking a blood meal from a vertebrate host, as observed in the Kamiti River virus, e.g., (Lutomiah et al., 2007). Horizonal transmission likely plays a part as well, although not via a vertebrate host as in arboviruses, but instead involving environmental transmission through contaminated water or plant material, through parasitic nematodes, or even venereally during mating (Agboli et al., 2019). There are several theories as to why ISVs are unable to replicate in vertebrate cells, and the mechanism for how ISVs cause infections in the mosquito is still unclear. The presence of an innate immune system and the increased body temperature in vertebrates are among the proposed mechanisms of host-restriction (Elrefaey et al., 2020).

### The role of ISVs in replication, transmission and pathogenic blocking strategy during superinfection with arboviruses

Insect-specific viruses have been shown to affect the ability of mosquitoes to disseminate arboviruses. Mosquitoes infected with ISVs may be less susceptible to arbovirus infection, reducing their ability to transmit these viruses to humans (Kent et al., 2010; Bolling et al., 2012, 2015; Hobson-Peters et al., 2013). Mosquitoes infected with the CxFV are for instance, less receptive to infection with the arbovirus West Nile virus (WNV). The mechanism behind this effect is not yet clear. It may be because of superinfection exclusion, also known as homologous interference, in which a cell or mosquito already infected with a virus cannot be infected with another virus of the same, or a closely related species (Folimonova, 2012; Hunter and Fusco, 2022). The phenomenon homologous interference may be due to many factors, such as competition for host receptors, the infected cell’s production of interferons or interferon-like substrates, or that the virus causing primary infection produces different substances like defective interfering viral genomes or trans-active proteases (Karpf et al., 1997; Harrison et al., 2024). Insect-specific flaviviruses have been observed to be especially prone to cause homologous interference, inhibiting *Flaviviridae* arbovirus infections *in vitro* and *in vivo* (Peterson et al., 2024).

It is difficult to draw conclusions about how ISVs interact with and affect the vector competence of mosquitoes for arboviruses as studies show contradictory results. The presence of previously unidentified or unknown ISVs could potentially obfuscate the results of infection studies, both when performed *in vitro* on insect cell lines, and *in vivo* when performed on mosquito colonies. As previously described, several ISVs were first discovered in established cell lines, suggesting that this has occurred previously. Some studies show that replication of arboviruses is inhibited when cells already are infected by ISVs, with CxFV inhibiting subsequent WNV infection, while another study shows that the same arbovirus is not affected at all by a primary infection with ISVs (Kent et al., 2010; Bolling et al., 2012; Hobson-Peters et al., 2013; Goenaga et al., 2020). The authors of a recent study suggest that the differences seen in these *in vitro* studies reflect the complex interactions between different viral strains and mosquito species, further increasing in complexity by the minute genetic and geographic variability necessarily introduced by *in vivo* study (Goenaga et al., 2020). Some findings suggest that the patterns may be further modulated by simultaneous infections of several insect-specific viruses (Schultz et al., 2018). Similar findings have also been seen in studies of the flavivirus Nhumirim virus in blocking WNV, Zika virus (ZIKV) and to a lesser extent, DENV-2 in C6/36 cells (Kenney et al., 2014; Romo et al., 2018). Other insect specific viruses where experimental co-infection has yielded similar results in recent years include Esprito Santo virus, Binjari virus (BINJV), Aripo virus (ARPV) and CFAV (Schultz et al., 2018; Auguste et al., 2021; White et al., 2021; Torres et al., 2022). More infection studies focusing on primary ISV infection and secondary arbovirus infection would be of great interest.

### ISV-based strategies for controlling arbovirus transmission and mosquito vectors

ISVs present a novel and promising approach for controlling mosquito populations and reducing the transmission of arboviruses. Due to their strict host restriction, infecting only insect cells and not vertebrates, ISVs provide a safe and environmentally friendly tool for vector control and pathogen interference (Blitvich and Firth, 2015; Bolling et al., 2015).

One of the key mechanisms by which ISVs can be applied is superinfection exclusion, where the presence of an established ISV infection in a mosquito inhibits the replication or transmission of subsequent arboviral infections. This phenomenon has been observed in various studies where ISVs reduced replication rates of medically important arboviruses such as DENV, ZIKV, and CHIKV (Kenney et al., 2014; Romo et al., 2018; Schultz et al., 2018). The interference may occur through competition for cellular machinery, induction of antiviral immune responses, or modulation of host gene expression, though the exact pathways are still being studied (Roundy et al., 2017).

ISVs also offer potential as biological delivery vectors. Advances in synthetic biology and molecular virology have raised the possibility of genetically modifying ISVs to express antiviral peptides or other inhibitory molecules. These engineered ISVs could be introduced into mosquito populations to block arbovirus replication or transmission without altering the mosquito genome directly, hence, offering a transgene-free alternative to traditional genetic modification approaches (Liu et al., 2024; Weng et al., 2024).

Furthermore, ISVs exhibit efficient vertical and horizontal transmission pathways. Vertical transmission from an infected female to her offspring ensures long-term persistence in mosquito populations, while horizontal transmission via venereal contact facilitates further spread among adults (Zhang et al., 2017; Wen et al., 2022; Peterson et al., 2024; Sharpe et al., 2024). These natural transmission routes may support the potential for self-sustaining, maintenance and population-wide establishment of ISVs in the field.

When integrated into broader vector control strategies, such as those involving *Wolbachia*—ISVs may play a complementary role. For example, both ISVs and *Wolbachia* exhibit pathogen-blocking capabilities, and their combined effects could enhance the overall suppression of arbovirus transmission (Moreira et al., 2009; Schnettler et al., 2016). The use of ISVs as part of a superinfection exclusion framework thus broadens the scope of biological control beyond single-strategy approaches.

However, challenges remain in realising the full potential of ISV-based approaches. Key considerations include the genetic stability of ISVs, risk of host-range expansion, ecological and environmental impact, as well as public acceptance and outlook in terms of reassurance of safety (Bolling et al., 2015; Roundy et al., 2017). Additionally, scalable production and release strategies will be necessary for successful implementation in endemic regions (Legros et al., 2012).

In essence, ISVs-based approaches hold significant promise for advancing the next generation of mosquito-borne disease control. Their specificity, safety, and potential for natural transmission make them attractive tools for integrated and sustainable public health interventions (Kenney et al., 2014; Blitvich and Firth, 2015).

### Superinfection exclusion in biological control: integrating *Wolbachia*-based approaches

The main reason for vector screening and surveillance is predicting and preventing disease outbreaks. There is a general lack of effective control strategies against the spread of arboviruses as the extermination of mosquitoes and larvae is not only expensive, but difficult, risking permanent ecological damage, as with the mass use of dichlorodiphenyltrichloroethane (DDT) in the mid 20th century (Tingström et al., 2016).

There is an ongoing discussion whether ISVs could be used as a potential method for biological control, like the endosymbiotic bacteria *Wolbachia* which infects several species of mosquitoes naturally. *Wolbachia* is both vertically and horizontally transmitted, and has been shown to persist in mosquito populations once introduced (Lilja et al., 2024). *Wolbachia* has been shown to reduce the vector competence for many arboviruses in different mosquito species. Likewise, ISVs could be used as a method of biological control, as ISVs are also naturally present in nature due to vertical transmission, and potentially cause homologous interference and increase the antiviral immune response of the mosquito (Blitvich and Firth, 2015; Vasilakis and Tesh, 2015).

When *Aedes aegypti*-mosquitoes are infected with the Wolbachia-strain *w*MelPop-CLA (*w*MelPop), it reduced the mosquitoes’ ability to be infected with DENV, CHIKV and the malaria parasite *Plasmodium* (McMeniman et al., 2009; Moreira et al., 2009; Bian et al., 2010; Kambris et al., 2010). This Wolbachia-strain represents an exception as most other strains are benign in their effects, having no effect on host lifespan, further research is required to elucidate whether the different strains of *Wolbachia* perform the same way in wild populations (McMeniman et al., 2009; Woolfit et al., 2013). The mechanism behind this is unclear, but available data suggests that an infection with *w*MelPop upregulates the immune-effector genes of the mosquito. Another theory is that *Wolbachia* and the arboviruses compete for the same vital cell components in the target cells (Moreira et al., 2009). *w*MelPop also reduces the lifespan of its host, roughly halving the lifespan of the fruit fly *Drosophilia melanogaster* and the mosquito *Aedes aegypti* (Woolfit et al., 2013). This effect on the longevity of the mosquito also affects its vector competence, as mosquito-borne viruses require a longer incubation period from the point that the virus infects the mosquito until it can be transmitted to a human. Only mosquitoes with a lifespan longer than the incubation period are therefore potentially infectious. Naturally, a shorter lifespan generally also allows for fewer opportunities for blood-feeding. Transmission of arboviruses to humans may therefore be reduced if mosquitoes have shorter lifespans, an effect further amplified by the upregulated immune system which reduces the risk of initial infection. Experimental infection of *Aedes aegypti* has however shown no negative effects on egg viability, allowing the strain to persist in wild populations, only decreasing the lifespan of the adult female vector (Kambris et al., 2009; Fox et al., 2024). When deployed in wild populations of *Aedes aegypti*, the *w*Mel *Wolbachia* has demonstrated a significant effect on preventing dengue infections endemically. The study demonstrated the high inter-mosquito transmission rate of *Wolbachia*, as 95.8% of mosquitoes in the population where the bacteria was introduced became infected. Although few studies have been carried out, factoring in differences in ecology, dengue strain and prevalence, as well as existing vector control strategies, the results are promising. Currently, such prevention programs are being facilitated globally by the World Mosquito Program (Fox et al., 2024).

This strengthens the value of this research project as ISVs somewhat comparable to *Wolbachia* could be used as potential methods of biological control through their effects on vector competence (Öhlund et al., 2019a). The use of ISVs for this purpose is not entirely without risk, as ISVs in theory could develop dual-host tropism, gaining the ability to infect not only insects, but humans as well. The possibility of insect specific viruses having the opposite effect, where a co-infection would in fact cause enhanced arbovirus replication cannot be exluded either. Studies have already shown such effects under certain circumstances when studying EILV and WNV co-infection (Guggemos et al., 2024; Joseph et al., 2024). In combination with the fact that viruses, unlike insecticides, can replicate in nature forming a potential snowball effect, care must be taken not to disrupt the ecology (Öhlund et al., 2019b).

### Evolution and tropism

Arboviruses may have developed from ISVs and through evolution developed dual-host tropism. There are, however, theories that some ISVs, especially in the genus *Alphavirus*, originally had dual-host tropism that they later lost (Erasmus and Weaver, 2017). A subgroup of ISVs known as lineage II insect-specific flaviviruses (LIN II ISVFs), which phylogenetically clustered closer to the vertebrate-infecting flaviviruses than ISVs, are suggested to have evolved from dual-host flaviviruses that lost their vertebrate tropism (Elrefaey et al., 2020; Harrison et al., 2020). More research is required to find out which of the two explains the tropism of arboviruses and ISVs (Nasar et al., 2015). The close genetic relationship between arboviruses and ISVs may be used to study the evolution from single-host to dual-host viruses. This information is of great importance, as it would explain how viruses develop and acquire new abilities, leading to increased pathogenicity (Öhlund et al., 2019b).

It remains unclear why ISVs have single-host tropism and lack the ability to infect vertebrate cells. A theory is that ISVs are inhibited by vertebrate cell-specific systems, parts of their innate immunity, among other reasons (Tree et al., 2016). A study of the insect-specific Eilat virus (EILV) found that it cannot infect vertebrate cells as the virus is blocked during its entry into the cell and RNA replication (Nasar et al., 2015). A chimeric virus including structural proteins of the dual host CHIKV (EILV/CHIKV) was, however, able to both bind and enter vertebrate cells, suggesting a host restriction in the entry step of the infection cycle (Erasmus and Weaver, 2017; Elrefaey et al., 2020). Other chimeras of ISVs have suggested later steps to be limiting in their respective infection cycles (Elrefaey et al., 2020; Hall et al., 2025). Some viruses have been characterized as intermediates between ISVs and arboviruses, such as the WNV-related flavivirus Rabensburg virus, first considered mosquito-specific, it was later revealed to infect avian cells, despite having never been isolated in an avian host. Rabensburg virus does not infect mammalian cells under physiological conditions and has not been isolated in any vertebrate host in nature (Ngo et al., 2019).

### Vaccine and drug development

There is no vaccine or specific treatment for many mosquito-borne diseases, such as Sindbis fever or Zika (Huang et al., 2023). Research suggests ISVs could have the potential of also aiding in the development of vaccines, mirroring the way in which the adenoviral vector vaccine was developed for COVID-19, though further study is required. Conventional live-attenuated vaccines, though often effective in offering long-lasting immunity, also carry the risk of reactogenic side effects. Attenuated vaccines lessen this risk, but through a trade-off of reduced immunogenicity. ISV vectored vaccines could potentially act as a method by which the advantages of both could be combined (Erasmus and Weaver, 2017; Harrison et al., 2024).

In a recent study, the ISV Aripo virus (ARPV) was used in a chimeric virus designated Aripo/Zika to produce a vaccine candidate for ZIKV, an arbovirus for which there is currently no available commercial vaccine (Porier et al., 2021; Peng et al., 2024). In a murine model, ARPV/ZIKV demonstrated no detectable adverse health effects after immunization, whilst being completely protected from morbidity after secondary infection. The innate host restriction imposed by using an ISV as a vaccine vector was seen as a key feature in the safety of the vaccine, as both ARPV and ARPV/ZIKV remained unable to replicate in vertebrate cells, even with high inoculation titers (Porier et al., 2021; Tanelus et al., 2023). Similar results were seen in a study where a cDNA-clone of the insect-specific EILV was designed to be a chimeric virus containing CHIKV-specific proteins, with the intended purpose of acting as a chikungunya vaccine (Erasmus and Weaver, 2017).

In a series of studies using chimeras of Binjari virus (BINJV), an ISF first detected in Australian *Aedes normanensis* in 2010, containing the PrM-E genes of ZIKV, WNW, DENV, YFV, and Japanese encephalitis virus (JEV), vaccine antigens used to produce a protective immune response in murine models (Harrison et al., 2021). In an immunodeficient murine model (IFNAR −/−), BINJ/ZIKV was shown to both induce an antibody response as well as result in a significant reduction in viremia and morbidity when challenged with wild-type ZIKV (Hobson-Peters et al., 2019; Hazlewood et al., 2022). Similar results were demonstrated for BINJ/JEV_NSW/22_ in IFNAR −/− mice (Harrison et al., 2024), BINJ/YFV_17D_ (Yan et al., 2020), as well as BINJ/WNV in CD1 mice (Vet et al., 2020),and BINJV/DENV2 in AG129 mice (Choo et al., 2021). In the latter two studies, complete protection against mortality when challenged with WNV_NY99_ and DENV2 were obtained, respectively. Of note is that the chimeric vaccines, except for the BINJ/YFV_17D_ vaccine, were able to produce this effect without requiring multiple doses. When comparing the chimera to their wild-type counterparts, the high degree of structural and antigenic authenticity was suspected to be a significant contributor to the immunogenic potency of chimeral vaccines; as the host range restriction of ISVs lifts the requirement for chemical inactivation or recombination, the structural integrity of the antigen is thus better maintained in the vaccine (Harrison et al., 2021). Similar chimeric vaccines have been proven safe and efficacious in non-human primates and pigs. Human trials have not yet been published (Hall et al., 2025). So far, similar vaccine vector studies have been performed on the insect-specific *alphavirus* Yada Yada virus, as well as the *orthoflaviviruses* YN15-283-02 and Chaoyang virus (Hall et al., 2025).

### Future perspectives

Insect-specific viruses offer an interesting field of study in the future, including whether ISVs, or other simultaneous infections like the *Wolbachia* bacteria, decrease the mosquito lifespan. More research in virus evolution is required to figure out if ISVs lost the ability to infect vertebrate cells, or if arboviruses gained it. Finding out how great the risk of ISVs developing dual-host tropism is would be of value in determining their safety in biological control. It remains unclear the exact mechanisms inhibiting ISVs from infecting vertebrate cells, and how ISVs affect and cause mosquito infection. More research into ISVs in vaccine and anti-viral development is required.

The use of ISVs as a means of inhibiting pathogens from infecting humans by using a virus that, through host restriction, does not itself infect humans, bears a striking similarity to the use of bacteriophages in the context of bacterial infections. Bacteriophages were widely used in the early 20th century, but later fell out of favor as penicillin was developed and widely adopted, except for in the former Soviet Union and Eastern Europe, where research continues (Strathdee et al., 2023). Much as with ISVs, widespread adoption has been halted due to safety concerns, as mutations could either enable the viruses to infect humans, or through transduction spread genes for antibiotic resistance or virulence factors. Both ISVs and bacteriophages are however abundant in nature, already exposing humans and animals continuously (Kakasis and Panitsa, 2019). These factors display an interesting potential of both ISVs and bacteriophages in medicine, as well as veterinary medicine and agriculture, where the specific tropism of these viruses pose an advantage over the environmental risks of the antibiotics and insecticides currently used (Strathdee et al., 2023).

The prospect of using ISVs in biological control, vaccine development, and antiviral therapy is compelling. Expanding knowledge about ISV distribution could significantly enhance outbreak preparedness. Hypothetically, ISVs should outnumber human-infecting viruses due to the high ratio of insect to vertebrate species (16:1) (Nasar et al., 2015). Confirming this will require broader surveillance and screening of mosquitoes for ISVs.

The global landscape of arbovirus transmission is rapidly changing. For example, in Europe, autochthonous cases of mosquito-borne diseases such as dengue and chikungunya, once rare, are now being reported (Laverdeur et al., 2024). This also applies to the mosquito species like *albopictus*, one of the main vectors for CHIKV, not originally endemic to Europe, but has spread in several countries around the Mediterranean Sea, and even further north in the Netherlands (Hubálek, 2008; Laverdeur et al., 2024). A reason for this mosquito species migrating further north, may be due to climate change and global warming (Hubálek, 2008). Demographical and social changes in a population may also play a part, as urbanization, population growth, and changes in land use all affect vector-host interaction (Tingström et al., 2016). Likewise, in Africa the spread of invasive mosquitoes for instance the invasive Asian urban mosquito-the *Anopheles stephensi* may increase the risk of o’nyong-nyong virus transmission in urban areas (Mutsaers et al., 2023). Humans travel mainly through containerized shipping, may provide a means through which adult mosquitoes, larvae, and eggs to potentially be introduced to new areas (Hubálek, 2008).

Given these developments, there is an urgent need for effective vector and virus control strategies. ISVs represent a high-potential avenue for future innovations in the control, prevention, and treatment of diseases caused by mosquito-borne arboviruses.
